# In vitro rumen fermentation kinetics, metabolite production, methane and substrate degradability of polyphenol rich plant leaves and their component complete feed blocks

**DOI:** 10.1186/s40781-018-0184-6

**Published:** 2018-11-09

**Authors:** Ganesh N. Aderao, A. Sahoo, R. S. Bhatt, P. K. Kumawat, Lalit Soni

**Affiliations:** 1 Animal Nutrition Division, ICAR- Indian Veterinary Research Institute, 243122, Izatnagar, UP India; 2 Division of Animal Nutrition Division, ICAR- Central Sheep and Wool Research Institute, Avikanagar, Rajasthan 304501 India

**Keywords:** Polyphenol, Methane production, Fermentation metabolites, degradability

## Abstract

**Background:**

This experiment aimed at assessing polyphenol-rich plant biomass to use in complete feed making for the feeding of ruminants.

**Methods:**

An in vitro ruminal evaluation of complete blocks (CFB) with (*Acacia nilotica*, *Ziziphus nummularia* leaves) and without (*Vigna sinensis* hay) polyphenol rich plant leaves was conducted by applying Menke’s in vitro gas production (IVGP) technique. A total of six substrates, viz. three forages and three CFBs were subjected to in vitro ruminal fermentation in glass syringes to assess gas and methane production, substrate degradability, and rumen fermentation metabolites.

**Results:**

Total polyphenol content (g/Kg) was 163 in *A. nilotica* compared to 52.5 in *Z. nummularia* with a contrasting difference in tannin fractions, higher hydrolysable tannins (HT) in the former (140.1 vs 2.8) and higher condensed (CT) tannins in the later (28.3 vs 7.9). The potential gas production was lower with a higher lag phase (L) in CT containing *Z. nummularia* and the component feed block. *A. nilotica* alone and as a constituent of CFB produced higher total gas but with lower methane while the partitioning factor (PF) was higher in *Z. nummularia* and its CFB. Substrate digestibility (both DM and OM) was lower (*P* < 0.001) in *Z. nummularia* compared to other forages and CFBs. The fermentation metabolites showed a different pattern for forages and their CFBs. The forages showed higher TCA precipitable N and lower acetate: propionate ratio in *Z. nummularia* while the related trend was found in CFB with *V. sinensis*. Total volatile fatty acid concentration was higher (*P* < 0.001) in *A. nilotica* leaves than *V. sinensis* hay and *Z. nummularia* leaves. It has implication on widening the forage resources and providing opportunity to use forage biomass rich in polyphenolic constituents in judicious proportion for reducing methane and enhancing green livestock production.

**Conclusion:**

Above all, higher substrate degradability, propionate production, lower methanogenesis in CFB with *A. nilotica* leaves may be considered useful. Nevertheless, CFB with *Z. nummularia* also proved its usefulness with higher TCA precipitable N and PF. It has implication on widening the forage resources and providing opportunity to use polyphenol-rich forage biomass for reducing methane and enhancing green livestock production.

## Background

Browses and trees invariably find their place in ruminant ration in most of the tropical countries. Polyphenols are one of the principal components of these feed resources, which have multiple di- and/or tri-hydroxyphenyl units to classify them as 1) condensed tannins, 2) hydrolysable tannins and 3) phlorotannins [1]. These constituents have huge structural diversity and their interactions with intrinsic (plant) and extrinsic (microbes) proteins have a profound effect on the outcome of expected positive effects on ruminal fermentation attributes and implications of their use in preparation of complete feed for ruminants. Exploitation of plant biomass rich in one or the other component polyphenols to manipulate rumen microbial population including methanogens and thereby harnessing positive rumen fermentation metabolites is considered a useful approach. Methane emission from enteric fermentation and manure management are some of the major sources of livestock GHG emission and several studies have been conducted to evaluate effect of various plant secondary  metabolites (PSM) for reducing methane production [2–4]. Various phytochemicals like saponins and tannins have been shown to modulate rumen fermentation favourably, and to inhibit methane production in the rumen [3, 4].

Leaves with high nutrient content, digestibility and low methane production can be used by marginal and landless farmers as supplementary feed resource for the feeding of small ruminants. Use of browse species containing secondary compounds as feed supplement for ruminants in many parts of the tropics is increasing in order to improve animal performance by diverting energy loss through methane towards production [5, 6]. The present study was thus aimed at evaluating the effect of polyphenol rich plant leaves alone or as component of complete feed block (CFB) on in vitro rumen fermentation attributes, methane production and substrate degradability.

## Methods

### Collection of forages and preparation of complete feed block

Conventional cowpea hay (*Vigna sinensis*) and polyphenolrich plants *Acacia nilotica* and *Ziziphus nummularia* leaves were harvested from the Agricultural Farm area of Central Sheep and Wool Research Institute, Avikanagar and dried in shade. Three different complete feed blocks (CFB) were prepared by incorporating 30 parts of these forages with concentrate mixture (65 parts) and molasses (5 parts). The composition (kg/100 kg) of concentrate mixture was maize 40, barley 36, groundnut cake 14, mustard cake 3, til cake 4, mineral mixture 2 and common salt 1. The molasses moiety was first mixed with the concentrate mixture, which followed subsequent mixing with the forages in a mechanical mixer. The composite mixture was then subjected to preparation of CFB by compressing at 5000 psi (351.5 kg/cm^2^) using a horizontal CFB making machine developed by NARP, Department of Agricultural Research and Education (DARE), New Delhi.

### Sample preparation and analysis

Representative samples of *A. nilotica* and *Z. nummularia* leaves, cowpea (*V. sinensis*) hay and CFB were collected and dried in hot air oven at 50–55 °C (~ 48 h) till constant weight. The dried samples were ground to pass 1 mm screen and stored in screw capped polycarbonate vials for further analysis. Chemical composition i.e. DM, EE, ash were analyzed by the methods of AOAC [7]. Nitrogen (N) content of the sample was estimated by distilling the digested sample in distillation unit (Gerhardt Vapodest 45 s, Germany) attached to auto titrator (TitroLine easy). Fiber fractions (i.e. neutral detergent fiber, NDF; acid detergent fiber, ADF) were determined by following the method of Van Soest et al [8]. Acid detergent residue was treated with 72% H_2_SO_4_ (*w*/w) and ashed for acid detergent lignin (ADL) estimation. Polyphenol fractions were analyzed by the methods described by Hagerman et al [9]. Folin-Ciocalteu method was used for the determination of total phenols [10]. The condensed tannins (CT) content was analyzed with the help of butanol- HCl reagent in the presence of ferric ammonium sulphate, and CT (g/Kg DM) is expressed as leucocyanidin equivalent.

$$ \mathrm{CT}\ \left(\mathrm{g}/\mathrm{Kg}\right)=\left({\mathrm{A}}_{550}\mathrm{nm}\times 78.26\times \mathrm{Dilution}\ \mathrm{factor}\right)/\left(\%\mathrm{Dry}\ \mathrm{matter}\right)\times 10\mathrm{a} $$

Where, A_550_ nm is absorbance at 550 nm.

For non-tannin phenolics (NTP) estimation, accurately weighed 100 mg PVPP, 1 mL each of distilled water and tannin-containing extract was transferred to a 15 mL test tube. Thereafter, the tubes were vortexed and kept at 4 °C for 15 min. and then centrifuged at 3000 rpm for 10 min to collect the supernatant, which was estimated for NTP by Folin-Ciocalteu method [10] and expressed as g/Kg DM. Total tannin phenols (TTP) were calculated as the difference between TP and NTP. Hydrolysable tannin (HT) was calculated as the difference between TTP and CT.

### In vitro gas production (IVGP) test

In vitro gas production (IVGP) technique of Menke et al [11] was followed for ruminal fermentation in glass syringes. Rumen liquor was collected from adult male rams being fed near maintenance in the morning (before feeding) with the help of stomach tube attached to a suction pump. It was transferred to a pre-warmed CO_2_ filled thermos, and immediately carried to the laboratory (max - 30 min). Rumen fluid from different rams collected was mixed in equal proportion, and filtered through four layered muslin cloth under continuous flushing of CO_2_ to maintain anaerobiosis. Oven-dried samples (200 mg) in triplicate were weighed into 100 mL calibrated glass syringes fitted with plungers. Syringes were filled with 30 mL of medium consisting of 10 mL rumen fluid and 20 mL buffer solution. Three blank syringes were also incubated with only 30 mL of the medium. The syringes were placed in hot water bath cum shaker maintained at 39 °C. Gas production (GP) was recorded after 2, 4, 6, 8, 10 12, 18, 24, 30, 36, 48 72 and 96 h of incubation. Net GP by each sample during the above mentioned period was calculated by subtracting the gas produced of the blank. The data so generated was processed as per Sigmastat Software (version 3.5) for calculating time to reach half asymptote (t^1/2^; h), potential GP (mg/200 mg substrate), rate constant (c) and lag phase (L; h). The GP kinetic parameters were calculated from the time dependent (0 to 96 h) in vitro cumulative GP data by applying single pool logistic model as depicted below. The assumptions were made that the rate of GP is proportional to both the accumulated microbial mass and to the amount of digestible substrate remaining [12].

$$ \mathrm{Y}\ \left(\mathrm{t}\right)=\mathrm{b}/\left[\right(1+\exp\ \left\{\right(2+4\mathrm{c}\ \left(\mathrm{L}\hbox{-} \mathrm{t}\right)\left\}\right] $$

Where, Y (t) = GP (mL) after time t, b = asymptotic value of the component (total potential GP, mL), c = specific rate of fermentation and L = lag time (the time axis intercept).

### Gas production and methane assay

After calculation of fermentation constants, two sets of samples each in triplicate were run simultaneously. In set one 200 mg and in set two 400 mg of oven dried samples weighed in to 100-mL glass syringes fitted with waxed plungers and were incubated with rumen buffer medium. In set one 30 mL and in set two 40 mL of the medium (with double strength buffer) was added as per the modified method of Menke and Steingass [13]. Samples were incubated in hot water bath cum shaker maintained at 39 °C up to 24 h and total GP was recorded. The gas sample from the first set was analyzed for methane concentration and fermentation was terminated by keeping the syringes in ice water. Methane (CH_4_) was analyzed by Gas Chromatograph (Model 1000, Series 011124002 of DANI make, Italy) using FID with PTV column. The temperature of injection port was 120 °C; column 50 °C; detector 120 °C. The flow rate of carrier gas (nitrogen) was 30 mL/min; hydrogen 30 mL/min; air 300 mL/min. The standard gas used for methane estimation composed of 99.998% methane (Sigma-Aldrich; Missouri, United States). Methane concentration was calculated by comparing the peak area of standard with samples. Methane production was calculated by applying the following equation.

$$ {\displaystyle \begin{array}{l}{\mathrm{CH}}_4\left(\mathrm{mL}\right)/100\ \mathrm{mg}\ \mathrm{digested}\ \mathrm{OM}=100\times \Big\{\left(\mathrm{GP}\ {\mathrm{t}}^{1/2}\times \left[{\mathrm{CH}}_4{\mathrm{t}}^{1/2}\right]\right)\ \\ {}-\left(\mathrm{GP}\ \mathrm{in}\ \mathrm{blank}\ {\mathrm{t}}^{1/2}\times \left[{\mathrm{CH}}_4\mathrm{of}\ \mathrm{blank}\ {\mathrm{t}}^{1/2}\right]\right)\Big\}/\mathrm{mg}\ \mathrm{digested}\ \mathrm{OM}\end{array}} $$

### Substrate degradability

The sample in set two (400 mg sample) syringes after incubation for 24 h was transferred to 600 mL spoutless beaker and 100 mL of neutral detergent solution was added after washing the in vitro syringes with the same solution (for ensuring quantitative transfer) and refluxed for 1 h as practiced during NDF assay. The samples were then filtered and washed through pre-weighed sintered glass crucibles (G-1) and the residue was dried in hot air oven at 100 °C for 24 h and weighed. The crucible with residue was incinerated in muffle furnace at 600 °C for 4 h and weighed next day after cooling. OM digestibility was calculated after necessary corrections for the blank samples.

### Statistical analysis

Analysis of variance was employed for data analysis following General Linear Model (GLM) procedure using statistical software SPSS (version 16). Tukey’s test was utilized to compare significant differences (*P* < 0.05) among the means for the two set of substrates (roughages and CFBs).

## Results

### Nutrient and polyphenolic composition

The nutrient composition of *V. sinensis* hay, *A. nilotica* leaves, *Z. nummularia* leaves revealed similar CP contents (140–145 g/Kg) with a varied fiber fractions, viz. comparatively high NDF, ADF and lignin in polyphenol-rich forages than *V. sinensis* hay (Table 1). The CFB’s containing aforementioned roughages followed a similar pattern for these nutrients. The polyphenolic fractions of these forages revealed low total phenol (TP) in *V. sinensis* (3.9 g/Kg) compared to *A. nilotica* (163 g/Kg) and *Z. nummularia* (52.5 g/Kg). *A. nilotica* had high TTP and HT whereas NTP and CT contents were higher in *Z. nummularia* (21.4 and 28.3 g/Kg). The polyphenolic composition of CFB followed a similar trend as it was for the component roughage moiety.

**Table 1:** Chemical and polyphenol constituents (g/kg dry matter) of roughage components and complete feed block (CFB)

Constituents	*Vigna sinensis* hay	*Acacia nilotica* leaves	*Ziziphus nummularia* leaves	CFB 1	CFB 2	CFB 3
DM	892	902	865	888	902	899
OM	882	876	874	909	896	902
Ash	118	124	126	91	104	97.8
CP	144	145	140	153	155	150
EE	25.4	25.7	32.1	52.1	53	54.8
NDF	608	695	657	374	400	389
ADF	451	468	473	252	257	259
ADL	108	124	196	60.4	65	86.6
Hemicellulose	157	227	184	122	143	130
Cellulose	343	344	277	192	192	172
Total phenols	3.9	163	52.5	2.7	45.2	16.3
Total tannin phenols	2.9	148	31.1	2.1	40.4	9.4
Non-tannin phenols	1	15	21.4	0.6	4.8	6.9
Condensed tannins	1.2	7.9	28.3	1.8	2.3	8.2
Hydrolysable tannins	1.7	140.1	2.8	0.3	38.1	1.2

*CFB1* Concentrate mixture + *Vigna sinensis* hay (70:30), *CFB2* Concentrate mixture + *Acacia nilotica* leaves (70:30), *CFB3* concentrate mixture + *Ziziphus nummularia* leaves (70:30)

### In vitro gas and methane production

In vitro fermentation constants, gas, methane production and substrate degradability of roughages revealed (Table 2) highest gas production (GP) in *A. nilotica* leaves (151 mL/g DM) followed by *V. sinensis* hay (137 mL/g DM) and *Z. nummularia* leaves (126 mL/g DM). Potential gas production (mL/200 mg DM) was significantly higher (*P* < 0.05) for *V. sinensis* hay (42.1) and *A. nilotica* (40.9) as compared to *Z. nummularia* leaves (37.9). The t½ was significantly lower (*P* < 0.05) in *A. nilotica* leaves as compared to *V. sinensis* hay and *Z. nummularia* leaves. Total methane produced per g of substrate was 28.4, 12.7 and 23.0 mL in these feed resources. DMD and OMD were higher in *A. nilotica* leaves (65.0, 68.8%) followed by *V. sinensis* hay (57.7 and 64.6%) and lowest value was observed in *Z. nummularia* leaves (54.9 and 60.2%). Significant (*P* < 0.05) difference were observed in methane production per g digestible DM/OM in different feed resources, viz. *V. sinensis* hay produced highest methane per unit of digestible DM and OM (49.1, 49.8 mL) followed by *Z. nummularia* leaves (41.9, 43.8 mL) and lowest in *A. nilotica* leaves (19.6, 22.3 mL). Amongst the CFBs, highest (*P* < 0.05) GP was recorded in CFB2 (233 mL/g DM) followed by CFB1 (221 mL/g DM) and CFB3 (184 mL/g DM) (Table 3). Potential gas production (mL/200 mg DM) was significantly lower (*P* < 0.05) in CFB3 as compared to CFB1 and CFB2. There was no significant difference observed for t½, rate constant and lag phase among CFBs. Methane produced per g of substrate was 50.8, 29.8 and 34.0 mL in CFBs.

**Table 2:** In vitro fermentation constants, degradability and methane production of different forages

Attributes	*Vigna sinensis* Hay	*Acacia nilotica* leaves	*Ziziphus nummularia* leaves	SEM	Significance (*P* value)
*Fermentation kinetics*					
Potential gas production (ml/200 mg DM)	42.1	40.9	37.9	0.926	0.021
Rate constant (c)	0.044	0.061	0.049	0.005	0.001
Half time (t^1/2^, h)	15.6	11.4	14.0	0.71	0.018
Lag phase(L, h)	1.90	3.33	5.37	1.16	0.012
*Degradability and methane production*					
DMD %	57.7^b^	65.0^a^	54.9^c^	0.31	< 0.001
OMD %	64.6^b^	68.8^a^	60.2^c^	0.59	< 0.001
Gas production (mL/g)	137^b^	151^a^	126^c^	3.1	< 0.001
Gas production (mL/g DDM)	237^a^	232^ab^	229^b^	2.5	0.024
Gas production (mL/g DOM)	240	250	239	5.2	0.541
Methane production (mL/g DM)	28.4^a^	12.7^c^	23.0^b^	0.95	< 0.001
Methane production (mL/g DDM)	49.1^a^	19.6^c^	41.9^b^	1.36	< 0.001
Methane production (mL/g OMD)	49.8^a^	22.3^c^	43.8^b^	0.70	< 0.001
Partitioning factor (g/mL)	4.16^b^	3.99^a^	4.18^b^	0.038	0.042

Means bearing different superscript (^a, b, c^) differ significantly

μm = maximum rate of GP (b × c) At the inflection point (Y = b/2), i.e. inflection occurs halfway to the maximum gas volume and thus, t_1/2_ = L + (μm × b/2)

**Table 3:** In vitro fermentation constants, degradability and methane production of complete feed block (CFB)

Attributes	CFB1	CFB2	CFB3	SEM	Significance (*P* value)
*Fermentation kinetics*					
Potential Gas Production (ml/200 mg DM)	61.9	61.4	55.4	1.096	0.031
Rate Constant (c)	0.059	0.064	0.060	0.005	0.082
Half time (t^1/2^ h)	11.7	10.8	11.6	1.12	1.121
Lag phase (h)	1.69	3.23	3.75	1.607	0.094
*Degradability and methane production*					
DMD %	79.5^a^	82.0^a^	74.0^c^	0.609	< 0.001
OMD %	81.8^b^	86.6^a^	80.3^c^	0.379	< 0.001
Gas production (mL/g)	221^a^	233^a^	184^b^	5.34	< 0.001
Gas production (mL/g DDM)	278	284	246	6.43	0.152
Gas production (mL/g DOM)	297^a^	300^a^	254^b^	6.45	0.012
Methane production (mL/g DM)	50.8^a^	29.8^c^	34.0^b^	1.64	< 0.001
Methane production (mL/g DDM)	63.9^a^	36.3^c^	45.9 ^b^	2.11	< 0.001
Methane production (mL/g DOM)	68.3^a^	38.3^c^	47.0^b^	0.809	< 0.001
Partitioning factor (g/mL)	3.36^a^	3.33^a^	3.94^b^	0.016	< 0.001

Means bearing different superscript (^a, b, c^) differ significantly

*CFB1* Concentrate mixture + *Vigna sinensis* hay (70:30), *CFB2* Concentrate mixture + *Acacia nilotica* leaves (70:30), *CFB3* Concentrate mixture + *Ziziphus nummularia* leaves (70:30)

### Substrate degradability and partitioning factor

Degradability of DM and OM was higher in CFB2 (82.0 and 86.6%) followed by CFB1 (79.5 and 81.8%) and lowest in CFB3 (75.6 and 80.3%) (Table 3). Similarly, significant (P < 0.05) difference were observed in methane production per unit digestible substrates, wherein CFB1 produced highest methane per unit of degradable DM and OM (63.9 and 68.3 mL) followed by CFB3 (47.5 and 47.0 mL) and lowest value (34.3 and 38.3 mL) was recorded in CFB2. The trend was in line with the roughage components (Table 2). The partitioning factor (PF) was low in *A. nilotica* leaves compared to other two roughages. However, the feed blocks containing these roughages showed a different pattern, being higher in CFB3 compared to CFB1 and CFB2.

### Rumen fermentation metabolites

*A. nilotica* leaves showed higher (*P* < 0.05) total N and NH_3_-N values than the other two substrates (Table 4). TCA precipitable N content was higher (*P* < 0.001) in *Z. nummularia* leaves than *A. nilotica* leaves, which was higher than *V. sinensis* hay. The VFA profile showed higher (*P* < 0.05) TVFA including acetate, propionate and other VFA fractions in *A. nilotica* leaves as compared to *Z. nummularia* and *V. sinensis* hay. The ratio of nonglucogenic to glucogenic VFA and acetate and propionate ratio were higher (*P* < 0.05) in *A. nilotica* leaves than the *Z. nummularia* leaves and *V. sinensis* hay.

**Table 4:** In vitro ruminal fermentation metabolites of different forages

Attributes	Roughage			SEM	Significance (*P* value)
	*Vigna sinensis* hay	*Acacia nilotica leaves*	*Ziziphus nummularia* leaves		
Total N (mg/dL)	47.3^c^	57.1^b^	60.5^a^	0.459	< 0.001
Ammonia N (mg/dL)	16.4^b^	17.3^a^	16.4^b^	0.209	0.046
TCA precipitable N (mg/dL)	15.7^b^	10.8^c^	21.8^a^	0.971	< 0.001
Acetic acid (mM/L)	26.3^b^	31.3^a^	11.1^c^	0.759	< 0.001
Propionic acid (mM/L)	5.31^a^	5.06^a^	2.60^b^	0.159	< 0.001
Isobutyric acid (mM/L)	0.88^a^	0.89^a^	0.26^b^	0.021	< 0.001
Butyric acid (mM/L)	2.89^a^	2.68^a^	2.28^b^	0.069	0.003
Isovaleric acid (mM/L)	0.75^a^	0.89^a^	0.24^b^	0.039	< 0.001
Valeric acid (mM/L)	0.37	0.34	0.64	0.103	0.137
Total VFA (mM/L)	36.5^b^	41.2^a^	17.1^c^	0.911	< 0.001
Branch-chain fatty acids (mM/L)	2.00^a^	2.13^a^	1.15^b^	0.119	0.002
Acetate: propionate ratio	4.96^b^	6.18^a^	4.25^c^	0.11	< 0.001
Non-glucogenic: glucogenic VFA ratio	4.66^b^	5.40^a^	4.47^b^	0.11	0.002

Means bearing different superscript (^a, b, c^) differ significantly

*TCA* Trichloroaceticic acid, *VFA* Volatile fatty acids

The pH was similar and total N (mg/dL) values ranged from 91.3 (CFB2) to 115.1 (CFB3) showing significant difference between the CFBs (Table 5). Conversely, the ammonia N concentration was higher (*P* < 0.05) in CFB2 than CFB1 and CFB3. The TCA precipitable N ranged from 44.6 in CFB2 to 78.8 in CFB1. Total VFA (mM/L) production was highest from CFB2 (48.7) followed by CFB1 (42.7) and CFB3 (25.4) and the concentration of acetate, propionate, BcFA and other FA followed a similar trend. The acetate: propionate ratio was higher (P < 0.05) in CFB3, but the ratio of nonglucogenic to glucogenic VFA was non-significantly different between the CFB types. The proportion of propionate was higher (P < 0.05) in CFB1 and CFB2 than CFB3.

**Table 5:** In vitro ruminal fermentation metabolites of complete feed block (CFB)

Attributes	Complete feed block				
	CFB1	CFB2	CFB3	SEM	Significance (*P* value)
Total N (mg/dL)	103^b^	91.3^c^	115^a^	1.52	< 0.001
Ammonia N (mg/dL)	18.1^b^	20.8^a^	17.8^b^	0.209	< 0.001
TCA precipitable N (mg/dL)	78.8^a^	44.6^c^	70.3^b^	1.67	< 0.001
Acetic acid (mM/L)	29.8^a^	33.6^a^	17.9^b^	1.73	< 0.001
Propionic acid (mM/L)	6.53^a^	7.44^a^	3.47^b^	0.351	< 0.001
Isobutyric acid (mM/L)	0.95^a^	1.00^a^	0.37^b^	0.029	< 0.001
Butyric acid (mM/L)	4.19^b^	5.03^a^	2.50^c^	0.179	< 0.001
Isovaleric acid (mM/L)	0.89^b^	1.09^a^	0.29^c^	0.029	< 0.001
Valeric acid (mM/L)	0.41^b^	0.45^b^	0.95^a^	0.041	< 0.001
Total VFA (mM/L)	42.8^a^	48.6^a^	25.5^b^	2.2	< 0.001
Branch-chain fatty acids (mM/L)	2.24^b^	2.54^a^	1.61^c^	0.063	< 0.001
Acetate: propionate ratio	4.54^b^	4.54^b^	5.13^a^	0.148	0.047
Nonglucogenic: glucogenic VFA ratio	4.59	4.64	4.82	0.108	0.38

Means bearing different superscript (^a, b, c^) differ significantly

*TCA* Trichloroaceticic acid, *VFA* Volatile fatty acids

*CFB1* Concentrate mixture + *Vigna sinensis* hay (70:30), *CFB2* Concentrate mixture + *Acacia nilotica* leaves (70:30), *CFB3* Concentrate mixture + *Ziziphus nummularia* leaves (70:30)

## Discussion

The nutrient composition of the three roughage moieties in the CFB, *V. sinensis* hay, *A. nilotica* leaves and *Z. nummularia* leaves are in line with the documented information [14, 15]. Total polyphenolic constituents in *A. nilotica* was higher than *Z. nummularia* leaves, but the tannin polyphenolic fractions (g/Kg DM) revealed a different pattern, the former was rich in HT (140) while the later was rich in CT (28.3). Similar polyphenolic composition in these tree leaves has also been reported earlier [16, 17] and in CFB, the level of polyphenolic constituents followed the trend as that in respective roughage source.

The three roughage substrates were degraded differently in the in vitro ruminal fermentation system. The substrate from *A. nilotica* leaves was degraded at the highest rate and it was not affected by the increased level of HT. On the contrary, *Z. nummularia* leaves showed a lower degradability value compared to conventional *V. sinensis* hay, due probably to the presence of higher CT and lignin content [3]. These values were comparable in *A. nilotica* leaves and *V. sinensis* hay. A similar trend in OM degradability between the substrates could also be attributed to this variation in chemical constituents. Alteration in substrate degradability due to differences in chemical composition is quite obvious [3, 16]. Consequently, t^½^ was lowest in *A. nilotica* leaves followed by *Z. nummularia* leaves and highest in *V. sinensis* hay. This was in direct relation with the degradability, as stated earlier by Sahoo et al [18] that halfway time to maximum gas volume is positively correlated with speed of microbial attachment and rate of degradation, which ultimately decides substrate degradability of forages. The rate constant was higher for *A. nilotica* and *Z. nummularia* leaves and lowest in *V. sinensis* hay. Different feedstuffs always demonstrate difference in rate constants during kinetic assay [19]. A high rate constant for *A. nilotica* leaves is justifiable from their higher GP. However, higher rate constant for *V. sinensis* hay may be ascribed to lowest NDF and high lignin.

A direct correlation does exist between substrate degradability, short-chain fatty acid production and IVGP [20]. Accordingly, *A. nilotica* leaves and CFB2 revealed higher GP. Expression of GP per unit digestible DM and OM registered higher values but the differences between the substrates narrowed down where all the three substrates had similar values because of variability in digestibility. This could be attributed to intrinsic higher CT [21] and lignin [3] content in *Z. nummularia* leaves compared to *V. sinensis* hay and *A. nilotica* leaves. These CT is having negative effect on GP because of their ability to interact with protein and fiber fractions, thereby reducing microbial enzymatic degradation (precipitate microbial enzymes) and microbial growth [3, 22, 23]. Highest DM and OM degradability in CFB2 followed by CFB1 and lowest in CFB3 was in line with CFBs with respective constituent roughages. Higher degradability was evidenced by higher GP in CFBs. Similar results were also observed by Bhatta et al. [23]. On similar line, low methane production by *A. nilotica* leaves may be attributed to its higher TTP compared to other forages. In tropical legumes, content of tannins and other secondary metabolites is higher that affects NDF digestibility and reduce methane production [24]. Methane inhibition activity observed in *Z. nummularia* leaves could thus be attributed to presence of CT. This alternatively produced positive fermentation pattern with better acetate: propionate and nonglucogenic: glucogenic VFA ratio [25]. Plants rich in tannins [25] and saponins [26] have potential for enhancing flow of microbial protein from rumen, increasing efficiency of feed utilization and decreasing methanogenesis. The CFB containing these plant biomass recorded a similar pattern and it was observed earlier too [3]. A lower methane emission for some shrubs (e.g. *Z. nummularia*) than other forages could thus be attributed to presence of various phytochemicals [3, 23, 27]. It has been suggested that the action of tannins on methanogenesis is attributed to direct inhibitory effects on methanogens [28] depending upon the chemical structure of tannins, and also indirectly by decreasing fiber degradation [3, 26, 29].

The data on fermentation metabolites was inconsistent in both roughage components and the CFBs. Variation in total N concentration in the digesta as against relatively similar N content in the three roughages was indicative of different fermentation behaviour effected by dissimilar phytochemicals. The concentration of ammonia N and TCA-precipitable N did not show any definite correlation. A higher ammonia N in *A. nilotica* leaves with intermediate total N concentration led to lower TCA-precipitable N compared to *V. sinensis* hay and *Z. nummularia* leaves. This reduction in TCA-precipitable N may be due to the harmful effects of higher HT and total polyphenol content on ruminal microbes responsible for metabolism of N fractions. Moreover, negative correlation between polyphenol content and TCA-precipitable N has also been reported [3]. Total VFA concentration was in line with substrate fermentation that degraded at higher rate to produce more gas. Similar correlation was observed earlier [3, 18, 20] and it is emphasized that the amount of gas produced from feeds depends largely upon chemical composition and rate and extent of degradability of feeds and production of VFA and their proportion. The proportion of acetate and other branched chain fatty acids (BcFA) that revealed better acetate: propionate ratio and nonglucogenic: glucogenic VFA ratio could be attributed to interaction of CT and other non-polyphenolic polymer lignin. Evidently, it was associated with higher proportion of propionate, butyrate and other BcFA in *Z. nummularia* leaves. A different trend in CFB with *Z. nummularia* leaves commensurate with higher CT and lignin content.

Preparation of total mixed ration based compact feed block involving crop residues, tree leaves and browses presents altogether a different substrate for ruminal microbial degradation that have different proportional distribution of soluble and insoluble carbohydrates, proteins and fats. Moreover, ruminal fermentation is a dynamic process involving production of fermentation metabolites, microbial synthesis and multiplication associated with gradual decline in substrate availability consequent upon its degradability and ensuing alteration in the total fermentation process. Consequently, the PF that demonstrates partitioning of nutrients in to microbial protein synthesis [20] may become a critical determinant to establish correlation between true substrate degradability and production of shortchain fatty acids, fermentable gas and methane production.

## Conclusion

The complete feed with HTrich *A. nilotica* leaves exhibited higher substrate degradability, propionate production and lower methanogenesis. On the other hand, CFB with CTrich *Z. nummularia* leaves produced lower fermentable gas, VFA with higher PF to emphasize its usefulness. This conflict could only be addressed by in vivo experiments. Nevertheless, this in vitro ruminal assessment of polyphenol-rich plant biomass and the CFBs could certainly elaborate the diverse effects on ruminal fermentation behaviour between polyphenolic constituents HT and CT.

Therefore, judicious incorporation of *A. nilotica* and *Z. nummularia* leaves in complete feeds would promise better ruminant production. Besides, inclusion of these leaves as part of total mixed ration would eventually help in delivering positive fermentation attributes by minimizing their antinutritional effects. Finally, polyphenol rich plant leaves can be used for strategic reduction of methane emission from the animals thereby diverting the energy for higher production with better feed efficiency.

## Abbreviations

ADF: Acid detergent fiber
ADL: Acid detergent lignin
BcFA: Branched-chain fatty acids
BW: Body weight
CFB: Complete feed block
CP: Crude protein
CRD: Complete randomized experimental design
CT: Condensed tannins
DM: Dry matter
DM: Dry matter
DWG: Daily weight gain
EE: Ether extract
FA: Fatty acids
FID: Flame-ionization detector
GP: Gas production
HCl: Hydrochloric acid
HT: Hydrolysable tannins
IVGP: In vitro gas production
ME: Metabolizable energy
N: Nitrogen
NDF: Neutral detergent fiber
NTP: Non-tannin phenols
OM: Organic matter
PTV: Programmable temperature vaporizer
TCA: Trichloro acetic acid
TP: Total phenols
TTP: Total tannin phenols
VFA: volatile fatty acids
